# Extrusion and Pancreatin Superdosing Modulate the Metabolic Impact of 20% *Chlorella vulgaris* Inclusion in Broiler Diets

**DOI:** 10.1111/jpn.70064

**Published:** 2026-04-26

**Authors:** Ana Rita Mendes, Cátia Falcão Martins, Maria Pinheiro Spínola, Daniela Filipa Pires Carvalho, Obete Madacussengua, Joana Inês Ferreira, Ana Maria Fontes, Rui Manuel Amaro Pinto, Miguel Pedro Mourato, Adam Davis, André Martinho de Almeida, Madalena Lordelo, José António Mestre Prates

**Affiliations:** ^1^ LEAF ‐ Linking Landscape, Environment, Agriculture and Food, Instituto Superior de Agronomia Universidade de Lisboa, Tapada da Ajuda Lisbon Portugal; ^2^ CIISA ‐ Centro de Investigação Interdisciplinar em Sanidade Animal, Faculdade de Medicina Veterinária Universidade de Lisboa, Av. da Universidade Técnica Lisbon Portugal; ^3^ AL4AnimalS ‐ Laboratório Associado para Ciência Animal e Veterinária Lisbon Portugal; ^4^ Associate Laboratory TERRA, Instituto Superior de Agronomia Universidade de Lisboa, Tapada da Ajuda Lisbon Portugal; ^5^ JCS, Dr. Joaquim Chaves, Laboratório de Análises Clínicas Carnaxide Portugal; ^6^ iMED.UL, Faculdade de Farmácia Universidade de Lisboa, Avenida Professor Gama Pinto Lisbon Portugal; ^7^ Department of Poultry Science, College of Agricultural and Environmental Sciences University of Georgia Athens Georgia USA

**Keywords:** *Chlorella vulgaris*, hepatic composition, microalga extrusion, pancreatin superdosing enzyme, plasma metabolite, poultry performance

## Abstract

Microalgae such as *Chlorella vulgaris* are gaining attention as sustainable and nutritionally valuable feed ingredients, with the potential to partially replace soybean meal in broiler diets. However, dietary inclusion of 20% *C. vulgaris* has been attempted, but negatively affected growth performance due to limited digestibility. This study examined the effects of including 20% *Chlorella vulgaris* (*C. vulgaris*) in broiler diets, either alone, extruded, or combined with 0.3% pancreatin supplementation, on blood parameters, plasma biochemistry, and liver composition. From day 7 to 35 of age, four dietary treatments were applied: a standard control diet (CTR), a diet with 20% *C. vulgaris* (CV), a diet with 20% *C. vulgaris* supplemented with 0.3% pancreatin (CVEN), and a diet containing 20% extruded *C. vulgaris* (CVEX). Findings showed that *C. vulgaris* dietary incorporation, regardless of treatment, led to a significant reduction in growth performance and an increase in specific plasma lipid parameters (*p* < 0.05) when compared with the CTR diet. However, in CVEN animals, such adverse effects were mitigated, resulting in improvements in final body weight and average body weight gain. Dietary inclusion of *C. vulgaris* modulated hepatic composition without affecting total hepatic lipid content. *Chlorella*‐based diets lowered PUFA/SFA and *n‐6/n‐3* ratios and increased specific fatty acids, particularly C16:0 and 20:5*n‐3*. Hepatic cholesterol and antioxidant‐related compounds, including β‐carotene, chlorophyll *a*–like pigments, α‐tocopherol, and, in untreated *C. vulgaris*, γ‐tocopherol and γ‐tocotrienol, were increased, and multivariate analysis clearly separated CTR diet‐fed birds from *Chlorella*‐fed groups. Multivariate analysis identified treatment‐related patterns in both blood and liver samples. PCA of plasma parameters clearly separated all dietary groups. Conversely, hepatic PCA distinguished control from *C. vulgaris* treatments, which clustered together regardless of processing or enzyme supplementation, indicating consistent liver responses to microalgae dietary inclusion. Overall, these findings indicate that a 20% inclusion level of *C. vulgaris* requires digestibility‐enhancing strategies to sustain broiler growth performance. Pancreatin supplementation effectively restored performance at this inclusion level to levels comparable to those of conventional maize–soy diets, highlighting the potential of enzyme‐treated *C. vulgaris* as a viable main ingredient that supports both production outcomes and health‐related attributes.

## 1 Introduction

The composition of poultry feed has evolved significantly over time, particularly with regard to protein sources. Traditionally reliant on animal‐based by‐products, such as fish meal, meat and bone meal, and blood meal, the European industry forcefully shifted toward plant‐derived alternatives due to bovine spongiform encephalopathy (BSE; mad cow disease), with soybean meal becoming the dominant protein source due to its high protein content, availability, and excellent amino acid profile. While soybean meal remains widely used, concerns about deforestation, land use, and competition with human food production have spurred interest in more sustainable and environmentally friendly options (Chaves et al. 2021), such as microalgae (Abdel‐Wareth et al. 2024; Costa et al. 2024a; Zhang et al. 2025). Among the microalgae, *Chlorella vulgaris* (*C. vulgaris*) stands out for its protein content and abundance of essential nutrients, including amino and fatty acids, carbohydrates, vitamins, pigments, and minerals. Furthermore, it has several functional advantages, such as antimicrobial, antioxidant, anti‐inflammatory and immunomodulatory effects (El‐Ghany 2020; Yongabi Anchang et al. 2016), that positively affect gut health and immunity of monogastric animals (El‐Ghany 2020; El‐Shall et al. 2023; Kang et al. 2013; Lee et al. 2023; Madigan‐Stretton et al. 2021; Martins et al. 2022; Merin et al. 2022; Mirzaie et al. 2018).

The rigid cell wall of *C. vulgaris*, which is composed of a complex matrix of polysaccharides, hemicellulose, and chitin‐like polymers, limits its nutrient digestibility in monogastric animals and may increase digesta viscosity at higher incorporation levels (Alfaia et al. 2021; Cabrol et al. 2022). This has restricted its dietary use mainly to low‐level supplementation (Gerken et al. 2013; Weber et al. 2022).

Mechanical and enzymatic pre‐treatments have been explored to overcome this limitation, with variable degrees of success. Mechanical approaches such as bead milling, sonication, extrusion, and high‐pressure homogenisation can disrupt the microalgae cell wall, increasing protein solubility and overall nutritional value (Alhattab et al. 2019; Chia et al. 2019; Costa et al. 2024a; Machado et al. 2022; Mendes et al. 2025; Spínola et al. 2023b; Van Nerom et al. 2024). Enzymatic supplementation, particularly with pancreatin, promotes protein hydrolysis and improves digestibility (Asare et al. 2022; Costa et al. 2024a). Moreover, enzyme superdosing has been associated with enhanced nutrient utilisation, gut health, and growth performance (Bromfield et al. 2021; Madigan‐Stretton et al. 2021). Incorporation of *C. vulgaris* and *Limnospira platensis* (Spirulina) in broilers has shown promising effects, often enhancing hepatic fatty acid profiles and antioxidant‐related compounds, although effects on growth performance are variable and may depend on inclusion level, pre‐treatment, or feeding strategy (Costa et al. 2024b; Fernandes et al. 2024; Mendes et al. 2025; Spínola et al. 2024). Inclusions of *C. vulgaris* (up to 10%) have been successfully applied in laying hen diets without adverse effects on egg production, feed intake, feed conversion ratio, and major egg quality parameters (Madacussengua et al. 2024).

Therefore, this study evaluates the effects of a high dietary inclusion of *C. vulgaris* (20%) introduced from 7 days of age on broiler growth performance, health status, blood metabolites, and hepatic lipid metabolism, including lipid, pigment, antioxidant, and mineral profiles. This experimental design builds upon previous works from our research team (Mendes et al. 2026), in which a 15% inclusion level introduced at 14 days of age was evaluated under feed extrusion and pancreatin superdosing. Here, we extend this nutritional and technological framework by investigating the effects of earlier dietary exposure, combined with a higher level of microalgae incorporation.

## 2 Material and Methods

### 2.1 Broilers Housing and Experimental Diets

The experiment used 120 1‐day‐old male Ross 308 broiler chicks (Pinto Valouro, Leiria, Portugal), with an average starting weight of 44.51  ±  0.52 g (mean ± standard deviation). Birds were randomly assigned to four experimental treatments (30 birds per treatment) and housed in 40 wire‐netted pens (56 × 56 × 50 cm), with three birds per pen and 10 pens (replicates) per treatment, the pen was considered the experimental unit. Broilers were reared for 35 days under controlled conditions at the School of Agriculture, University of Lisbon. Environmental parameters, such as temperature and ventilation, were regularly monitored in accordance with standardised protocols (Mendes et al. 2025). Mash, feed, and water were provided *ad libitum*.

From hatching until day 7, all broiler chicks received a standard mash‐form starter diet composed of maize and soybean meal. Beginning on day 7 and continuing through day 35, birds were assigned to one of four grower experimental diets, all provided daily in mash form and available *ad libitum*: (1) a control diet based on maize and soybean meal (CTR); (2) a diet incorporating 20% *C. vulgaris* powder sourced from Allmicroalgae (Pataias, Portugal) (CV); (3) the same *C. vulgaris* inclusion with the addition of 0.3% pancreatin extract (CVEN; Merck, Darmstadt, Germany); and (4) a diet containing 20% extruded *C. vulgaris* (CVEX; Sparos, Olhão, Portugal) as described by Mendes et al. (2025) and Spínola et al. (2023a). To comply with the 3Rs principles (Replacement, Reduction, and Refinement) in animal research, each pen housed three chicks, with ten pens assigned to each dietary treatment.

A detailed breakdown of ingredients and additives used in each diet is provided in Table 1.

**Table 1:** Formulation of broiler experimental diets^a^ (day 7–35; % as‐fed basis).

Ingredients	CTR	CV	CVEN	CVEX
Maize	54.45	50.77	50.77	51.52
Soybean meal 44%	37.65	24.90	24.90	24.15
Sunflower oil	4.29	1.48	1.48	1.47
Sodium chloride	0.400	0.160	0.160	0.170
Calcium carbonate	1.08	1.48	1.48	1.50
Dicalcium phosphate	1.60	0.650	0.650	0.650
DL‐Methionine	0.130	0.120	0.120	0.120
L‐Lysine	0	0.040	0.040	0.020
Vitamin‐mineral premix^b^	0.400	0.400	0.400	0.400
*Chlorella vulgaris*	0	20.0	20.0	0
*Chlorella vulgaris* extruded	0	0	0	20.0
Porcine pancreatin	0	0	0.30	0

*Note:* Vitamins: vitamin A (10,000 IU), vitamin D3 (2400 IU), vitamin E (30 mg), vitamin K3 (2 mg), vitamin B1 (2 mg), vitamin B2 (4 mg), vitamin B6 (2 mg), vitamin B12 (0.02 mg), folic acid (1 mg), nicotinic acid (25 mg), pantothenic acid (10 mg); Minerals: copper (8 mg), iron (50 mg), iodine (0.7 mg), manganese (60 mg), selenium (0.18 mg), zinc (40 mg).

^a^ CTR control's group diet based on maize and soybean meal; CV 20% *Chlorella vulgaris* incorporation; CVEN 20% *Chlorella vulgaris* incorporation and 0.3% pancreatin enzyme; CVEX 20% extruded *Chlorella vulgaris*.

^b^ Premix composition per kg of feed (as‐fed basis).

### 2.2 Nutritional Analysis of *Chlorella vulgaris* and Experimental Diets

Table 2 presents the proximate composition of *C. vulgaris*, in both its gently spray‐dried (raw) and extruded forms, as well as the experimental diets. Proximate composition was determined according to AOAC (2000) protocols, with samples ground to pass a 1 mm sieve and analysed in triplicate. Gross energy was determined using an adiabatic bomb calorimeter (Parr 6400, Parr Instrument Company, Moline, IL, USA).

**Table 2:** Nutritional and proximate analysis, estimated and determined values, of *Chlorella vulgaris* and extruded *Chlorella vulgaris* and of broiler experimental diets^a^ (day 7–35).

Item	Spray‐dried *C. vulgaris*	Extruded *C. vulgaris*	CTR	CV	CVEN	CVEX
***Gross energy, kcal/kg as dry matter***	4742	4648	4060	4062	4050	4033
***Estimated amino acids availability, %***						
Lysine	—	—	1.15	1.15	1.15	1.15
Methionine	—	—	0.459	0.455	0.455	0.456
Threonine	—	—	0.795	0.726	0.726	0.727
TSAA^b^	—	—	0.727	0.602	0.602	0.594
***Proximate composition, % as dry matter***						
Dry matter	96.2	96.3	88.3	89.7	89.9	89.3
Crude protein	28.3	30.8	22.3	23.4	22.8	22.0
Crude fat	6.74	6.46	8.31	7.28	6.04	6.22
Ash	4.18	4.22	6.54	5.78	5.91	5.89
***Fatty acid profile, % total fatty acids***						
14:0	0.156	0.173	0.068	0.081	0.091	0.091
16:0	22.3	22.8	9.42	13.2	13.5	13.5
16:1*c*9	0.180	0.226	0.117	0.147	0.147	0.154
18:0	1.40	1.52	2.97	2.53	2.45	2.41
18:1*c*9	11.5	13.5	29.6	26.4	25.7	26.6
18:1*c*11	0.492	0.513	0.815	0.733	0.751	0.755
18:2*n‐6*	31.3	29.7	50.6	44.4	44.5	44.4
18:3*n‐3*	8.44	7.92	1.14	2.82	2.86	2.63
20:0	0.203	0.216	0.358	0.370	0.368	0.371
20:1*c*11	0.137	0.143	0.193	0.203	0.194	0.203
***Diterpene profile, µg/g***						
α‐Tocopherol	54.3	50.3	29.3	31.8	24.2	30.4
α‐Tocotrienol	—	—	2.24	1.61	1.93	2.00
β‐Tocopherol	—	—	0.646	0.377	0.397	0.418
γ‐Tocopherol + β‐tocotrienol	—	—	9.49	9.09	9.13	9.54
γ‐Tocotrienol	—	—	10.0	9.17	9.97	10.0
δ‐Tocopherol	—	—	0.880	0.647	0.620	0.698
***Pigments, µg/g***						
β‐Carotene	59.9	27.7	1.21	13.4	11.2	7.60
Chlorophyll *a* c	640	491	0.970	108	105	75.5
Chlorophyll *b* d	147	35.5	1.09	21.6	19.0	9.33
Total chlorophylls^e^	787	527	2.05	130	124	84.9
Total carotenoids^f^	335	169	3.63	53.2	48.0	23.7
Total chlorophylls + carotenoids^g^	1122	696	5.69	183	172	109
***Mineral profile, g/kg dry matter***						
Calcium	3.91	3.54	13.3	12.6	10.6	12.0
Copper	0.007	0.007	0.010	0.011	0.006	0.012
Iron	0.113	0.103	0.152	0.162	0.128	0.160
Magnesium	1.20	1.16	2.14	1.84	1.68	1.84
Manganese	0.044	0.042	0.079	0.092	0.094	0.083
Phosphorous	11.9	11.9	7.53	7.46	7.13	7.23
Potassium	7.05	7.10	11.3	9.43	9.26	9.45
Sulphur	3.75	3.69	3.20	3.22	2.96	3.14
Sodium	5.18	4.85	2.41	2.97	2.71	3.16
Zinc	0.178	0.165	0.087	0.118	0.108	0.121

^a^ CTR control's group diet based on maize and soybean meal; CV 20% *C. vulgaris* incorporation; CVEN 20% *C. vulgaris* incorporation and 0.3% pancreatin enzyme; CVEX 20% extruded *C. vulgaris*.

^b^ TSAA, total sulphur amino acids.

^c^ Ca = chlorophyll a, calculated as 11.24 × A662 − 2.04 × A645.

^d^ Cb = chlorophyll b, calculated as 20.13 × A645 − 4.19 × A662.

^e^ Ca + b = total chlorophylls, calculated as 7.05 × A662 + 18.09 × A645.

^f^ Cx + c = total carotenoids, calculated as (1000 × A470 − 1.90 × Ca − 63.14 × Cb)/214.

^g^ Ccc = total pigment content, calculated as Ca + b + Cx + c.

The fatty acid composition was determined by direct transesterification and analysed following Costa et al. (2024b), using gas chromatography with flame ionisation detection (GC‐FID; HP7890A, Hewlett‐Packard, Avondale, PA, USA), equipped with a Supelcowax® 10 capillary column (30 m × 0.20 mm i.d., 0.20 μm film thickness; Supelco, Bellefonte, PA, USA). Fatty acid methyl esters (FAMEs) were identified using a 37‐component standard mixture and quantified with C21:0 as an internal standard.

β‐carotene and diterpenes were assessed by high‐performance liquid chromatography (HPLC) according to Alfaia et al. (2021). In summary, samples (0.1 g) were saponified, extracted, and separated on a normal‐phase silica column. β‐carotene was detected using a UV‐visible photodiode array detector (λ = 450 nm), while tocopherols and tocotrienols were detected by fluorescence (excitation λ = 295 nm; emission λ = 325 nm). Quantification was based on external calibration curves with analytical standards in duplicate.

Pigment profiling followed the methods described by Pestana et al. (2020) and Hynstova et al. (2018). Samples (0.1 g) were extracted with 5 mL of acetone, homogenised in the dark, and centrifuged. The supernatant was analysed by UV‐Vis spectrophotometry (Genesys 150, Thermo Scientific, Waltham, MA, USA) at 662 nm and 645 nm for chlorophylls a and b, and 470 nm for total carotenoids. Pigment concentrations were determined using specific absorbance‐based equations adapted from Hynstova et al. (2018) and presented in Table 2.

The mineral composition was determined as described by Ribeiro et al. (2020). In summary, the lyophilised sample (0.3 g) was subjected to acid digestion with concentrated nitric and hydrochloric acids, followed by hydrogen peroxide oxidation. Digestion was performed at 95°C using a DigiPREP MS heating block (SCP Science, Baie d'Urfe, QC, Canada). After dilution and filtration, mineral concentrations were quantified by Inductively Coupled Plasma Optical Emission Spectrometry (ICP‐OES; iCAP 7200 Duo, Thermo Scientific, Waltham, MA, USA) using external calibration curves.

### 2.3 Broilers Slaughtering, Data, and Sample Collection

Each week, the birds, the feed offered, and the feed refusals (remaining in the feeders) were weighed. Average daily feed intake (ADFI) was calculated as the difference between the total feed offered and weekly refusals, divided by seven to obtain the daily average. Average daily gain (ADG) was calculated based on body weight changes over time and expressed as a daily average. Final body weight (FBW) and feed conversion ratio (FCR) were also determined.

One 35‐day‐old broiler of median pen weight was selected from each replicate (pen), resulting in 10 birds per treatment (one bird per pen) being slaughtered. Birds were electrically stunned and manually exsanguinated, with blood samples collected directly into Sarstedt tubes (Nümbrecht, Germany). Samples were centrifuged at 1500 × g for 15 min at 4°C to separate plasma, which was then stored at 2°C–8°C and subsequently analysed. Liver tissues were also collected, finely chopped, vacuum‐sealed, and preserved at –20°C until further analysis.

### 2.4 Blood Cells and Plasma Metabolites Analysis

Haemoglobin content and blood cell quantification, including erythrocytes, leucocytes, and thrombocytes, were performed using Sysmex XN‐10 automated haematology analysers (Sysmex Corporation, Kobe, Japan), following the procedures described by dos Santos Madeira et al. (2021). Erythrocyte counts were obtained via impedance‐based detection, employing hydrodynamic focusing to ensure accurate cell separation. For leucocyte differentials, blood smears were prepared and stained using the May‐Grünwald‐Giemsa method, then manually assessed to determine the percentage distribution of white blood cell types. Haemoglobin levels were assessed photometrically, utilising sodium lauryl sulphate as the lytic and chromogenic agent.

Plasma metabolite analysis encompassed measurements of glucose, urea, creatinine, total protein, and lipid profile components, including high‐density lipoprotein (HDL) cholesterol, low‐density lipoprotein (LDL) cholesterol, total cholesterol, and triacylglycerols (TAG). Additionally, enzymatic activities of alanine aminotransferase (ALT; EC 2.6.1.2), alkaline phosphatase (ALP; EC 3.1.3.1), aspartate aminotransferase (AST; EC 2.6.1.1), and γ‐glutamyltransferase (GGT; EC 2.3.2.13) were assessed using a Modular Hitachi analytical platform (Roche Diagnostics, Mannheim, Germany) in conjunction with commercial reagents (Roche Diagnostics, Basel, Switzerland). Very‐low‐density lipoprotein (VLDL) cholesterol and total lipid concentrations were estimated using established equations: VLDL‐cholesterol = TAG/5, as proposed by Friedewald et al. (1972), and total lipids = (total cholesterol × 1.12) + (TAG × 1.33) + 148, according to Covaci et al. (2006).

C‐reactive protein (CRP) levels were quantified via immunoturbidimetric analysis (Roche Diagnostics, Meylan, France) and key electrolytes ‐ sodium (Na⁺), potassium (K⁺), and chloride (Cl⁻) ‐ were quantified using the indirect potentiometric method.

### 2.5 Lipid and Fatty Acid Composition Analysis in Liver Tissue

Total lipids were extracted in duplicate from lyophilised liver samples using a dichloromethane–methanol mixture (2:1, v/v), following the protocol originally described by Folch et al. (1957). Lipid quantification was performed gravimetrically using a rotary evaporator (RV‐10, IKA Werke, Staufen, Germany) after solvent removal.

For fatty acid profiling, lipid extracts were converted to fatty acid methyl esters (FAME) via sequential alkaline and acid‐catalysed transesterification. FAMEs were analysed by gas chromatography (GC System 7890 A, Agilent Technologies, California, USA) equipped with a Supelcowax® 10 capillary column (30 m × 0.20 mm ID, 0.20 μm film thickness; Supelco, Bellefonte, PA, USA) equipped with a flame ionisation detector (FID), following the method described by Pestana et al. (2020).

### 2.6 Determination of Total Cholesterol, Diterpenes and β‐Carotene in Liver Tissue

Liver samples (0.75 g each, in duplicate) were analysed for total cholesterol, diterpenes, and β‐carotene content using a direct saponification method, followed by a single *n*‐hexane extraction and HPLC, following the protocol previously applied to the experimental diets, as outlined by Alfaia et al. (2021), adapted from Mestre Prates et al. (2006).

For hepatic pigment extraction, 1.5 g of liver tissue was homogenised in 3 mL of acetone under low‐light conditions for 1 min. The homogenate was then centrifuged at 3000 rpm for 5 min at 4°C. The absorbance of the resulting supernatant was recorded using a UV‐Visible spectrophotometer (Genesys 150, Thermo Fisher Scientific, Waltham, MA, USA). Pigment concentrations were calculated using the same equations and reference values employed for *C. vulgaris*, extruded *C. vulgaris* and dietary samples (see Section 2.2), based on Hynstova et al. (2018).

### 2.7 Determination of Mineral Profile in Liver Tissue

The hepatic mineral composition was assessed using the same validated methodology previously described for *C. vulgaris* and the experimental diets.

### 2.8 Statistical Analysis

Data were analysed using a completely randomised design within the Generalised Linear Model (GLM) framework, as implemented in SAS software (SAS Institute Inc., Cary, NC, USA). The statistical model included dietary treatment as the sole fixed effect. The experimental unit varied by the variable assessed: for growth performance metrics (FBW, ADG, ADFI, FCR), the pen (comprising three birds) was the experimental unit, whereas individual animals were the unit of analysis for plasma metabolites and detailed liver measurements.

Prior to analysis, assumptions of homogeneity of variances and normality were evaluated using Levene's test and the Shapiro–Wilk test, respectively. When significant differences were detected, multiple comparisons among least squares means were performed using the Tukey–Kramer adjustment (PDIFF option). Results are expressed as means with their corresponding standard error of the mean (SEM), and statistical significance was declared at α = 0.05.

To explore associations between plasma biomarkers and hepatic parameters, principal component analysis (PCA) was carried out using SPSS Statistics (version 27.0; IBM Corp., Armonk, NY, USA).

## 3 Results

### 3.1 Effects of Experimental Diets on Key Performance Indicators

Four dietary treatments were evaluated: a control diet (CTR), a diet with 20% *C. vulgaris* (CV), a diet with 20% *C. vulgaris* supplemented with 0.3% pancreatin (CVEN), and a diet containing 20% extruded *C. vulgaris* (CVEX). The pen was considered the experimental unit for all performance parameters.

**Figure 1:**
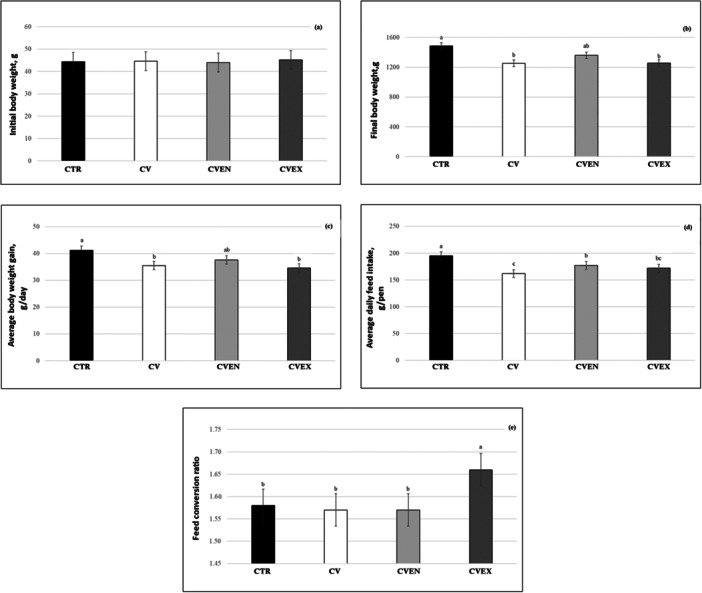
Growth performance of broilers fed experimental diets: (a) Initial body weight; (b) Final body weight; (c) Average body weight gain; (d) Average daily feed intake; (e) Feed conversion ratio. Diets: a maize‐soybean meal control diet (CTR), a diet containing 20% *C. vulgaris* (CV), a diet containing 20% *C. vulgaris* supplemented with 0.3% pancreatin (CVEN), or a diet containing 20% extruded *C. vulgaris* (CVEX). Values are presented as mean and SEM (standard error of the mean). ^a,b,c^ Different superscripts indicate a significant difference (*p* < 0.05).

Figure 1 presents an overview of the growth performance outcomes for broilers fed four different diets. FBW was significantly greater in birds fed the CTR diet compared to those on the CV and CVEX diets (*p* < 0.001). However, broilers fed the CVEN diet had an FBW value that was not statistically different from that of the CTR or the other two diets (CV and CVEX). A similar pattern of statistical significance was observed for ADG (*p* < 0.001) among the dietary treatments.

All *C. vulgaris diets* resulted in significantly lower feed intake than in the CTR group (*p* < 0.001). For broilers fed the CVEX treatment, FCR was greater (*p* < 0.001) than it was in the broilers from the other dietary treatments. Notably, mortality remained at 0% throughout the trial across all dietary treatments.

### 3.2 Effects of Experimental Diets on Blood Cells and Plasma Metabolites

Table 3 shows the haematological and plasma biochemical profiles of animals fed the experimental diets. No significant differences were observed among diets for white blood cells, granulocytes, lymphocytes, red blood cells, haemoglobin, thrombocytes, haematocrit (Hct), mean corpuscular volume (MCV), mean corpuscular haemoglobin (MCH), or mean corpuscular haemoglobin concentration (MCHC) (*p* > 0.05).

**Table 3:** Haematological variables and plasma metabolite levels of broilers fed with four different diets^a^.

Item	CTR^b^	CV^b^	CVEN^b^	CVEX^b^	SEM	*p*‐value
White blood cells (×10^9^/L)	7.03	7.86	7.92	7.23	1.221	0.939
Granulocytes (%)	48.3	47.4	48.0	48.8	1.63	0.938
Lymphocytes (%)	50.7	51.6	51.0	51.2	1.63	0.938
Red blood cells (×10^12^/L)	3.87	3.86	3.92	3.77	0.220	0.966
Haemoglobin (g/dL)	11.8	11.9	11.7	11.7	0.48	0.992
Thrombocytes (×10^9^/L)	24.2	24.2	23.6	21.5	2.61	0.857
Hct (%)	37.1	37.6	38.3	36.6	2.04	0.948
MCV (fL)	96.0	97.4	97.6	97.4	0.47	0.084
MCH (pg)	30.9	31.0	29.9	31.3	0.98	0.788
MCHC (g/dL)	32.1	31.8	30.7	32.1	0.93	0.671
Glucose (mg/dL)	249^b^	263^a^	260^a^	264^a^	1.7	< 0.001
Urea (mg/dL)	2.75^a^	1.16^c^	1.93^b^	1.86^b^	0.168	< 0.001
Creatinine (mg/dL)	0.007	0.008	0.007	0.007	0.0007	0.741
Cholesterol (mg/dL)	112^b^	126^a^	109^b^	130^a^	2.0	< 0.001
LDL‐cholesterol (mg/dL)	13.2^b^	18.3^ab^	13.5^b^	21.7^a^	1.44	< 0.001
HDL‐cholesterol (mg/dL)	81.9^a^	84.0^a^	70.2^b^	80.5^a^	1.38	< 0.001
VLDL‐cholesterol (mg/dL)^c^	9.66^b^	16.2^a^	16.7^a^	17.9^a^	0.48	< 0.001
Triacylglycerols (mg/dL)	48.3^b^	81.1^a^	83.3^a^	89.7^a^	2.42	< 0.001
Total lipids (mg/dL)^d^	423^c^	484^a^	451^b^	500^a^	5.6	< 0.001
ALT (U/L)	1.90	2.00	1.60	1.50	0.246	0.4334
AST (U/L)	323^a^	170^c^	212^b^	187^bc^	8.0	< 0.001
GGT (U/L)	19.2^ab^	22.4^a^	20.5^a^	17.0^b^	0.87	0.001
ALP (U/L)	2551^b^	3488^a^	2262^b^	2090^b^	1.0	< 0.001
Total protein (g/dL)	2.53^a^	2.44^a^	2.18^b^	2.48^a^	0.054	< 0.001
C‐reactive protein (mg/dL)	0.016^ab^	0.012^b^	0.014^b^	0.021^a^	0.0015	< 0.001
Na^+^ (mEq/L)	151^a^	145^b^	146^b^	151^a^	1.1	< 0.001
K^+^ (mEq/L)	5.95^b^	7.04^a^	6.11^b^	6.73^a^	0.087	< 0.001
Cl^−^ (mEq/L)	113^ab^	110^b^	111^b^	115^a^	1.1	0.007

*Note*: ^a,b,c^Different superscripts denote significant differences within each row (*p* < 0.05).

Abbreviations: ALT, alanine aminotransferase (EC 2.6.1.2); ALP, alkaline phosphatase (EC 3.1.3.1); AST, aspartate aminotransferase (EC. 2.6.1.1); Cl^‐^, chloride; GGT, γ‐glutamyltransferase (EC 2.3.2.13); Hct, haematocrit; HDL, high‐density lipoproteins; K^+^, potassium; LDL, low‐density lipoproteins; MCH, mean corpuscular haemoglobin; MCHC, mean corpuscular haemoglobin concentration; MCV, ‐ mean corpuscular volume; Na^+^, sodium; VLDL, very low‐density lipoproteins.

^a^ SEM, standard error of the mean; *n* = 10 replicate pens with one bird selected per pen.

^b^ CTR control's group diet based on maize and soybean meal; CV 20% *C. vulgaris* incorporation; CVEN 20% *C. vulgaris* incorporation and 0.3% pancreatin enzyme; CVEX 20% extruded *C. vulgaris*.

^c^ VLDL‐Cholesterol = 1/5 [triacylglycerols], as described by Friedewald et al. (1972).

^d^ Total lipids = [total cholesterol] × 1.12 + [triacylglycerols] × 1.33 + 148, as described by Covaci et al. (2006).

Blood glucose levels were significantly higher in all *C. vulgaris*–based treatments (CV, CVEN, CVEX) compared to the CTR group (*p* < 0.001). Urea levels in the blood were significantly lower in all *C. vulgaris* diets, particularly in the CV diet, which showed a 58% reduction compared to the CTR animals (*p* < 0.001).

Significant differences were observed in the plasma cholesterol profile among the dietary treatments. Total cholesterol levels were higher in birds fed CV and CVEX diets compared to CTR and CVEN (*p* < 0.001). A similar trend was observed for HDL‐cholesterol (*p* < 0.001). VLDL‐cholesterol and TAG increased in all *C. vulgaris* diets relative to the CTR (*p* < 0.001). Regarding LDL‐cholesterol, inclusion of 20% extruded *C. vulgaris* (CVEX) resulted in higher levels compared to the CTR (*p* < 0.001). Total lipids were highest in the CV and CVEX groups, intermediate in CVEN, and lowest in the CTR group (*p* < 0.001).

Plasma ALP levels increased drastically in the CV group compared to the CTR (*p* < 0.001), while ALT remained similar across all treatments (*p* = 0.4334). AST decreased in all microalga‐treatments relative to the CTR (*p* < 0.001). GGT levels were similar to the control in all *C. vulgaris* treatments, although the CVEX diet showed slightly lower GGT values (*p* = 0.001).

Total plasma protein was significantly lower in birds fed the CVEN diet compared to all other treatments (*p* < 0.001). The plasma CRP levels were affected by diet (*p* < 0.001), with the highest values in birds fed CVEX, the lowest in CV and CVEN, and intermediate values in the CTR diet.

Significant differences were also observed among treatments in the blood concentration of the electrolytes sodium (Na⁺), potassium (K⁺), and chloride (Cl⁻) (*p* = 0.007 for Cl^‐^; *p* < 0.001 for both K⁺ and Na⁺).

### 3.3 Effects of Experimental Diets on Liver Lipid Content, Cholesterol, and Fatty Acid Composition

Table 4 presents the hepatic total lipid content, total cholesterol, and fatty acid composition of broilers fed the four experimental diets. Total hepatic lipid levels did not differ significantly among the dietary groups (*p* = 0.483). In contrast, total hepatic cholesterol was significantly increased in all *C. vulgaris* treatments (*p* < 0.001).

**Table 4:** Hepatic lipid content, cholesterol levels, and fatty acid profile of broilers fed with four different diets^a^.

Item	CTR^b^	CV^b^	CVEN^b^	CVEX^b^	SEM	*p*‐value
***Total lipids, g/100 g***	2.70	2.15	2.08	2.26	0.305	0.483
***Total cholesterol, mg/g***	2.68^b^	3.26^a^	3.39^a^	3.26^a^	0.118	< 0.001
***Fatty acids composition, g/100 g of fatty acids***						
12:0	0.024	0.038	0.036	0.026	0.0049	0.142
14:0	0.175	0.153	0.148	0.156	0.0141	0.560
15:0	0.064^a^	0.050^ab^	0.048^b^	0.049^ab^	0.0043	0.032
16:0	19.3^b^	22.1^a^	21.9^a^	21.9^a^	0.51	0.001
16:1*c*7	0.372	0.302	0.296	0.313	0.0221	0.084
16:1*c*9	0.255^b^	0.733^ab^	0.588^b^	1.09^a^	0.1317	< 0.001
17:0	0.307^a^	0.211^b^	0.224^b^	0.197^b^	0.0201	0.002
17:1*c*9	0.046^ab^	0.085^a^	0.042^b^	0.059^ab^	0.0105	0.036
18:0	22.5	21.3	22.8	21.1	0.76	0.320
18:1*c*9	10.4	13.1	10.5	13.7	1.07	0.072
18:1*c*11	0.978^b^	1.16^ab^	1.05^ab^	1.28^a^	0.0720	0.028
18:2*n‐6*	16.7	16.8	19.3	11.2	2.46	0.134
18:3*n‐6*	4.71	1.96	0.041	7.56	2.3620	0.131
18:3*n‐3*	0.076^b^	0.186^a^	0.178^a^	0.174^a^	0.0122	< 0.001
20:0	0.066^b^	0.084^a^	0.074^ab^	0.073^ab^	0.0044	0.040
20:1*c*11	0.183^b^	0.320^a^	0.255^ab^	0.304^a^	0.0206	< 0.001
20:2*n‐6*	1.17^a^	0.825^b^	0.872^b^	0.765^b^	0.0606	< 0.001
20:3*n‐6*	1.64^b^	2.26^a^	2.26^a^	2.22^a^	0.119	0.002
20:4*n‐6*	13.9	12.2	13.1	12.0	0.53	0.055
20:3*n‐3*	0.033^b^	0.061^a^	0.061^a^	0.059^a^	0.0057	0.005
20:5*n‐3*	0.030^b^	0.205^a^	0.216^a^	0.237^a^	0.0137	< 0.001
22:0	0.072	0.080	0.069	0.070	0.0059	0.545
C22:1*n‐9*	0.022^b^	0.043^a^	0.040^a^	0.038^a^	0.0031	0.001
C22:2*n‐6*	0.043^b^	0.102^a^	0.064^ab^	0.062^ab^	0.0116	0.012
C22:5*n‐3*	0.404^b^	0.550^ab^	0.596^a^	0.520^ab^	0.0499	0.048
C22:6*n‐3*	0.537^b^	0.907^ab^	1.08^a^	0.771^ab^	0.1196	0.022
C23:0	0.056	0.090	0.066	0.060	0.0129	0.293
Others	5.94^a^	4.13^b^	4.20^b^	4.00^b^	0.255	< 0.001
Σ SFA^c^	42.5	44.1	45.3	43.6	0.80	0.119
Σ MUFA^d^	12.3	15.7	12.8	16.8	1.24	0.069
Σ PUFA^e^	39.3^a^	36.1^b^	37.7^ab^	35.6^b^	0.80	0.011
Σ *n‐6* PUFA^f^	38.2^a^	34.1^b^	35.5^ab^	33.8^b^	0.71	< 0.001
Σ *n‐3* PUFA^g^	1.07^b^	1.91^a^	2.13^a^	1.76^a^	0.142	< 0.001
PUFA/SFA	0.929^a^	0.822^b^	0.832^b^	0.816^b^	0.0253	0.012
*n‐6*/*n‐3*	38.2^a^	18.2^b^	17.3^b^	20.6^b^	1.94	< 0.001

*Note*: ^a,b^Different superscripts denote significant differences within each row (*p* < 0.05).

^a^ SEM, standard error of the mean; *n* = 10 replicate pens with one bird selected per pen.

^b^ CTR control's group diet based on maize and soybean meal; CV 20% *C. vulgaris* incorporation; CVEN 20% *C. vulgaris* incorporation and 0.3% pancreatin enzyme; CVEX 20% extruded *C. vulgaris*.

^c^ SFA (saturated fatty acids) ‐ Sum (12:0, 14:0, 15:0, 16:0, 17:0, 18:0, 20:0, 22:0, 23:0).

^d^ MUFA (monounsaturated fatty acids) ‐ Sum (16:1*c*7, 16:1*c*9, 17:1*c*9, 18:1*c*9, 18:1*c*11, 20:1*c*11, 22:1*n*‐9).

^e^ PUFA (polyunsaturated fatty acids) ‐ Sum (18:2*n‐6*, 18:3*n‐6*, 18:3*n‐3*, 20:2*n‐6*, 20:3*n‐6*, 20:4*n‐6*, 20:3*n‐3*, 20:5*n‐3*, 22:2*n‐6*; 22:5*n‐3*, 22:6*n‐3*).

^f^ *n‐6* PUFA ‐ Sum (18:2*n‐6*, 18:3*n‐6*, 20:2*n‐6*, 20:3*n‐6*, 20:4*n‐6;* 22:2*n‐6*).

^g^ *n‐3* PUFA ‐ Sum (18:3*n‐3*, 20:3*n‐3*, 20:5*n‐3*, 22:5*n‐3*, 22:6*n‐3*).

Dietary treatments significantly affected the hepatic fatty acid profile. The most abundant fatty acids in the liver (> 10% of total fatty acids) in the CTR broilers, in descending order, were 18:0, 16:0, 18:2*n‐6*, 20:4*n‐6*, and 18:1*c*9. Compared to CTR, broilers from the CV, CVEN, and CVEX groups showed higher levels of 16:0 (*p* = 0.001), which became the predominant fatty acid in CV and CVEX broilers. The inclusion of dietary *C. vulgaris* resulted in additional significant alterations, with decreases in 17:0 and 20:2*n‐6* (*p* < 0.003), and increases in 18:3*n‐3*, 20:3*n‐3*, 20:5*n‐3*, and 22:1n‐9 (*p* < 0.005) relative to CTR. Both extrusion and enzyme supplementation (CVEX and CVEN) moderated the hepatic content of specific fatty acids, such as 20:0 and 22:2*n‐6* (*p* = 0.040 and *p* = 0.012, respectively).

Significant differences were observed in the sum and ratios of hepatic fatty acids, specifically for polyunsaturated fatty acid (PUFA) (*p* = 0.011), *n‐3* PUFA, and *n‐6* PUFA (*p* < 0.001 for both). The *n‐3* PUFA levels were significantly higher in all *C. vulgaris* treatments, while PUFA and *n‐6* PUFA were lower in CV and CVEX treatments. Regarding the *n‐6*/*n‐3* ratio, all *C. vulgaris* treatments had a reduction of more than 45% compared with the CTR group (*p* < 0.001). Similarly, the hepatic PUFA/SFA ratio was also lower than in the control broilers relative to the broilers fed diets containing *C. vulgaris* (*p* = 0.012).

### 3.4 Effects of Experimental Diets on Liver Diterpenes and Pigments

Table 5 presents the results of the hepatic diterpene and pigment profiles of broilers fed different experimental diets. Significant differences were observed among treatments in the levels of α‐tocopherol, γ‐tocotrienol, and the combined levels of γ‐tocopherol and β‐tocotrienol among the diets (*p* < 0.001, *p* = 0.023, and *p* = 0.005, respectively).

**Table 5:** Hepatic vitamin E homologues and pigment concentrations of broilers fed with four different diets^a^.

Item	CTR^b^	CV^b^	CVEN^b^	CVEX^b^	SEM	*p*‐value
***Diterpene profile, µg/g***						
α‐Tocopherol	11.3^b^	20.5^a^	18.7^a^	16.7^a^	1.20	< 0.001
γ‐Tocopherol + β‐tocotrienol	0.271^b^	0.480^a^	0.345^ab^	0.410^ab^	0.0400	0.005
α‐Tocotrienol	0.028	0.029	0.036	0.031	0.0031	0.327
β‐Tocopherol	0.033	0.041	0.039	0.033	0.0029	0.134
γ‐Tocotrienol	0.095^b^	0.160^a^	0.103^ab^	0.092^b^	0.0170	0.023
***Pigments, µg/100 g***						
β‐Carotene	0.742^b^	15.7^a^	12.9^a^	16.8^a^	1.367	< 0.001
Chlorophyll *a*‐like pigments^c^	7.22^c^	21.9^ab^	23.9^a^	17.6^b^	1.476	< 0.001
Chlorophyll *b*‐like pigments^d^	12.6	18.4	19.8	13.8	2.44	0.126
Total chlorophyll like pigments^e^	19.8^b^	40.3^a^	43.7^a^	31.4^ab^	3.67	< 0.001
Total carotenoids^f^	184^b^	1861^a^	1806^a^	1725^a^	76.6	< 0.001
Total chlorophylls like pigments+carotenoids^g^	204^b^	1901^a^	1849^a^	1757^a^	75.7	< 0.001

*Note*: ^a,b,c^Different superscripts denote significant differences within each row (*p* < 0.05).

^a^ SEM, standard error of the mean; *n* = 10 replicate pens with one bird selected per pen.

^b^ CTR control's group diet based on maize and soybean meal; CV 20% *C. vulgaris* incorporation; CVEN 20% *C. vulgaris* incorporation and 0.3% pancreatin enzyme; CVEX 20% extruded *C. vulgaris*.

^c^ Ca = 11.24 × A_662_ ‐ 2.04 × A_645_.

^d^ Cb = 0.13 × A_645_ ‐ 4.19 × A_662_.

^e^ Ca + b = 7.05 × A_662_ + 18.09 × A_645_.

^f^ Cx + c = (1000 × A_470_ ‐ 1.90 × Ca ‐ 63.14 × Cb)/214.

^g^ (Ca + b) + (Cx + c).

All *C. vulgaris* groups exhibited higher α‐tocopherol levels when compared to the CTR treatment. The CV broilers had the highest γ‐tocotrienol levels, exceeding those of the CTR and CVEX broilers, whereas the CVEN broilers showed intermediate levels. For γ‐tocopherol and β‐tocotrienol combined levels were greatest in the CV group (0.480 µg/g) and lower in the control group (0.271 µg/g).

Regarding hepatic pigments, total carotenoid concentrations increased markedly in all broilers fed *C. vulgaris* diets (*p* < 0.001), representing approximately a 10‐fold elevation compared with the CTR broilers. A similar trend was observed for the combined total of chlorophyll‐like pigments and carotenoids (*p* < 0.001). The hepatic β‐carotene and chlorophyll *a*–like pigments concentrations were substantially greater in all *C. vulgaris*‐fed broilers compared to the CTR broilers (*p* < 0.001). In contrast, no significant differences were detected among treatments for chlorophyll *b*–like pigments (*p* = 0.126).

### 3.5 Effects of Experimental Diets on Liver Minerals

Table 6 outlines the hepatic mineral profile of broilers fed various experimental diets, detailing the concentrations of both macro‐ and microminerals. Hepatic phosphorus and copper levels were significantly greater in the CVEN broilers compared to the CTR broilers. All *C. vulgaris* treatments (CV, CVEN, CVEX) showed higher manganese levels than the control broilers (*p* < 0.001).

**Table 6:** Hepatic mineral content of broilers fed with four different diets^a^.

Item	CTR^b^	CV^b^	CVEN^b^	CVEX^b^	SEM	*p*‐value
***Macrominerals, mg/100 g***						
Calcium (Ca)	20.5	20.4	20.2	20.0	0.51	0.884
Magnesium (Mg)	24.8	24.2	24.4	24.2	0.34	0.494
Phosphorus (P)	305^b^	310^ab^	329^a^	320^ab^	5.6	0.032
Potassium (K)	338	332	338	334	4.5	0.715
Sodium (Na)	87.9	77.2	89.8	76.0	6.36	0.301
Sulphur (S)	200	190	202	189	5.2	0.202
Total macrominerals	976	954	1004	963	16.3	0.162
***Microminerals, mg/100 g***						
Copper (Cu)	0.273^b^	0.300^ab^	0.315^a^	0.297^ab^	0.0089	0.019
Iron (Fe)	18.7	12.5	12.2	14.3	2.66	0.303
Manganese (Mn)	0.271^b^	0.348^a^	0.381^a^	0.372^a^	0.0183	< 0.001
Zinc (Zn)	2.20	2.19	2.47	2.28	0.091	0.138
Total microminerals	21.5	15.4	15.4	17.3	2.66	0.337
***Total macro‐ and microminerals***	997	969	1020	981	16.7	0.181

*Note*: ^a,b^ Different superscripts denote significant differences within each row (*p* < 0.05).

^a^ SEM, standard error of the mean; *n* = 10 replicate pens with one bird selected per pen.

^b^ CTR control's group diet based on maize and soybean meal; CV 20% *C. vulgaris* incorporation; CVEN 20% *C. vulgaris* incorporation and 0.3% pancreatin enzyme; CVEX 20% extruded *C. vulgaris*.

The sum of macro, the sum of micro, and the combined level of both showed no significant differences among dietary treatments (*p* > 0.05).

### 3.6 Principal Component Analysis of Broilers' Plasma and Liver Composition

**Figure 2:**
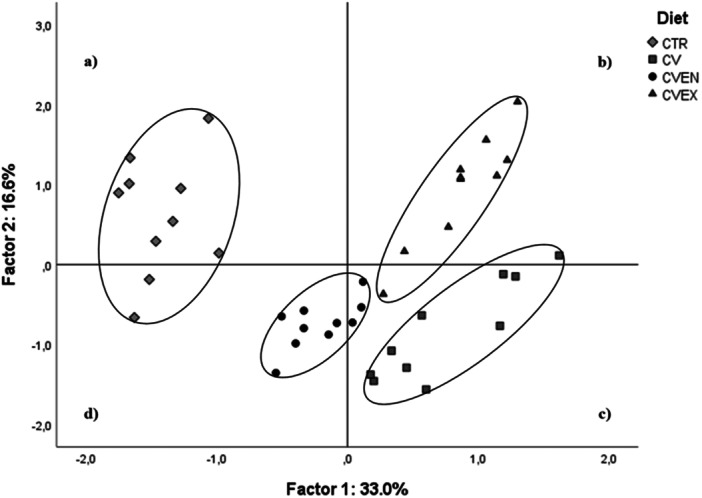
Principal component analysis (PCA) of plasma metabolites in broilers fed experimental diets: a maize‐soybean meal control diet (CTR), a diet containing 20% *C. vulgaris* (CV), a diet containing 20% *C. vulgaris* supplemented with 0.3% pancreatin (CVEN), or a diet containing 20% extruded *C. vulgaris* (CVEX).

**Figure 3:**
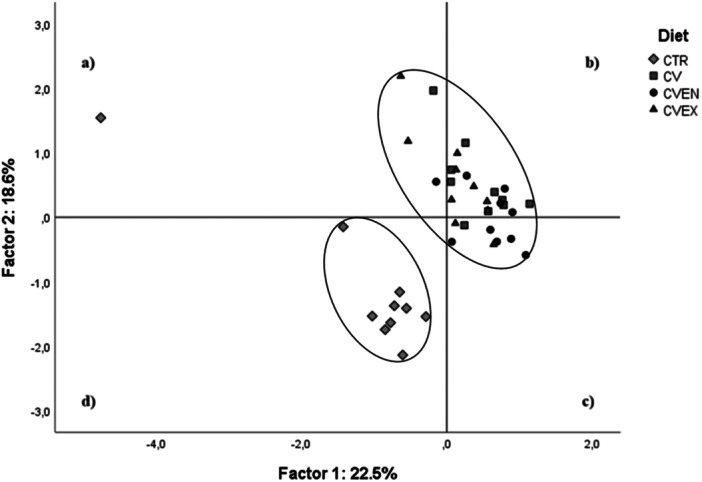
Principal component analysis (PCA) of hepatic metabolites in broilers fed experimental diets: a maize‐soybean meal control diet (CTR), a diet containing 20% *C. vulgaris* (CV), a diet containing 20% *C. vulgaris* supplemented with 0.3% pancreatin (CVEN), or a diet containing 20% extruded *C. vulgaris* (CVEX).

Principal Component Analysis (PCA) was applied to broiler plasma metabolite (Figure 2) and hepatic compound profiles (Figure 3), revealing clear separations among experimental diets.

Figure 2 presents the PCA biplot of broiler plasma metabolites, revealing clear separations among dietary treatments. Factor 1 and Factor 2 explained 33.0% and 16.6% of the variance, respectively (49.6% total). The CTR, CV, CVEN, and CVEX treatments formed distinct clusters, highlighting diet‐induced differences. Total lipids (0.949), triglycerides (0.883), and VLDL‐cholesterol (0.883) contributed most to Factor 1, whereas sodium (Na⁺; 0.874), chloride (Cl⁻; 0.651), and total protein (0.624) were the main contributors to Factor 2 (Table 7).

**Table 7:** Contribution of plasma metabolites and hepatic chemical composition to the first and second principal components, ranked by descending loading on Factor 1.

Plasma metabolites			Liver		
*Variables*	*Factor 1*	*Factor 2*	*Variables*	*Factor 1*	*Factor 2*
Total lipids	0.949	0.183	Cholesterol	0.847	0.006
Triglycerides	0.883	−0.076	14:0	−0.779	0.251
VLDL‐Cholesterol	0.883	−0.076	18:1*c*11	−0.774	0.277
Aspartate Aminotransferase (AST)	−0.854	0.305	16:1*c*7	−0.741	−0.037
Potassium (K^+^)	0.782	−0.071	Total carotenoids	0.701	0.600
Cholesterol	0.781	0.351	20:3*n‐6*	0.682	0.290
**Glucose**	0.751	−0.007	Phosphorous (P)	0.680	−0.298
**LDL‐Cholesterol**	0.720	0.465	**Total lipids**	−0.677	0.310
**Urea**	−0.591	0.550	Chlorophyll *a‐*like pigments	0.674	0.383
Alanine Aminotransferase (ALT)	−0.077	−0.049	Manganese (Mn)	0.659	0.209
Sodium (Na⁺)	−0.133	0.874	22:0	−0.629	0.225
Chloride (Cl^−^)	0.088	0.651	β‐Carotene	0.598	0.553
Total protein	0.043	0.624	22:5*n‐3*	0.578	−0.057
C‐reactive protein	0.155	0.558	α‐Tocopherol	0.565	0.486
γ ‐glutamyltransferase	0.020	−0.371	Copper (Cu)	0.552	0.066
Alkaline Phosphatase (ALP)	0.194	−0.333	20:3*n‐3*	0.545	0.262
**Creatinine**	−0.024	−0.221	α‐Tocotrienol	0.500	−0.144
**HDL‐Cholesterol**	0.053	0.102	22:1*n‐9*	−0.494	0.424
			22:6*n‐3*	0.471	−0.024
			Zinc (Zn)	0.427	−0.374
			22:2*n‐3*	0.394	0.283
			20:0	0.328	0.063
			Chlorophyll *b*‐like pigments	0.277	0.103
			23:0	0.257	−0.010
			18:3*n‐6*	−0.112	0.037
			16:1*c*9	−0.086	0.776
			20:5*n‐3*	0.128	0.774
			20:2*n‐6*	0.252	−0.742
			17:0	−0.172	−0.721
			Sulphur (S)	0.469	−0.700
			20:4*n‐6*	0.612	−0.672
			18:3*n‐3*	0.348	0.658
			Magnesium (Mg)	0.324	−0.626
			18:0	0.556	−0.611
			18:1*c*9	0.094	0.595
			20:1*c*11	0.353	0.565
			Potassium (K)	0.309	−0.505
			15:0	−0.455	−0.486
			16:0	0.281	0.483
			γ‐Tocopherol	0.400	0.466
			14:1*c*9	−0.300	0.454
			Iron (Fe)	−0.120	−0.410
			17:1*c*9	0.042	0.402
			Sodium (Na)	0.074	−0.307
			18:2*n‐6*	0.011	−0.176
			12:0	−0.026	0.056
			Calcium (Ca)	0.014	0.051

Figure 3 shows the PCA biplot of hepatic metabolites in broilers. Factor 1 and Factor 2 accounted for 22.5% and 18.6% of the variance (41.1% total). The CTR diet was clearly separated from the *C. vulgaris*‐based diets, which partially overlapped. Cholesterol (0.847), fatty acid 14:0 (−0.779), and 18:1c11 (−0.774) were the main contributors to Factor 1, while 16:1c9 (0.776), 20:5*n*‐3 (0.774), and 20:2*n*‐6 (−0.742) were the main contributors to Factor 2 (Table 7).

## 4 Discussion

This study provides novel in vivo evidence on the feasibility and limitations of incorporating *C. vulgaris* by using four dietary treatments (CTR, CV, CVEN, and CVEX) at a high dietary inclusion level (20%) in broiler diets from days 7 to day 35. Although *C. vulgaris* is widely recognised as a valuable feed supplement (Dinalli et al. 2024; Kang et al. 2013; Mendes et al. 2024a, 2024b), its application at levels higher than 5% has presented inconsistent results and can benefit from future studies. Recent studies using microalgae meal as a partial replacement for soybean meal have highlighted that its effects on performance are highly dependent on inclusion level, timing of dietary introduction, and feeding strategy (e.g., withdrawal before the finishing phase), with lower inclusion levels generally showing more consistent results (Zampiga et al. 2023; Zampiga et al. 2024). The present results contribute to this body of knowledge by evaluating the impact of a 20% inclusion and assessing whether processing strategies, namely extrusion and pancreatin supplementation, can mitigate potential constraints associated with high incorporation rates (Çelekli et al. 2024; Vandamme et al. 2013).

The dietary inclusion of *C. vulgaris* at 20% allowed a substantial reduction in soybean meal incorporation, decreasing from 37.65% in the CTR diet to 24.90% or less in the *C. vulgaris* diets. However, such a high inclusion was associated with lower FBW in birds fed the CV and CVEX diets compared to CTR, while CVEN birds did not differ from any of the other treatments. These results should, however, be interpreted with caution, as a larger sample size could improve the statistical power to detect differences in performance parameters. This is in line with previous reports cautioning against inclusion levels above 10% due to reduced digestibility and growth performance (Alfaia et al. 2021; Becker 2007; Cabrol et al. 2022). In contrast, Mendes et al. (2025) reported no detrimental effects at a 15% inclusion level, suggesting that the impaired performance observed in the present study is primarily attributable to the higher inclusion rate and age at dietary introduction. Regarding processing effects, extrusion did not improve performance at the 20% inclusion level, with CVEX animals exhibiting a higher FCR. These findings contrast with those of Mendes et al. (2025), suggesting that extrusion may enhance performance only within a physiological tolerance threshold. However, pancreatin superdosing mitigated the reduction in FBW, as CVEN animals showed values comparable to the other treatments. This supports the hypothesis that, at higher inclusion levels, enzymatic superdosing supplementation may be more effective than extrusion in overcoming nutritional and digestibility constraints associated to *C. vulgaris* dietary inclusion.

Concerning haematological parameters, no significant differences were observed, indicating that a 20% inclusion of *C. vulgaris* did not compromise physiological status. Red blood cell counts and haemoglobin concentrations were unaffected, and plasma glucose increased in all *C. vulgaris* diets, consistent with results by Mendes et al. (2026), but still remaining within the physiological range reported for broiler chickens (Vale et al. 2019). In terms of nitrogen metabolism, urea decreased in CV‐fed birds while creatinine remained unchanged, indicating improved nitrogen utilisation without renal impairment, similar to Mendes et al. (2026) and Spínola et al. (2024).

Hepatic enzymes showed mild adaptive responses: ALP, AST, and ALT patterns suggest adaptation without hepatocellular damage (Coelho et al. 2021; Mendes et al. 2026), while hepatic GGT activity varied inconsistently with diet, possibly reflecting differences in hepatobiliary function or oxidative metabolism (Shepherd 1989; Xing et al. 2022).

The absence of major alterations in haematological and hepatic damage markers suggests that the reduced growth performance observed in broilers fed 20% *C. vulgaris* was not driven by systemic physiological distress, but rather by nutritional and digestibility constraints limiting nutrient availability for tissue accretion.

All *C. vulgaris* diets increased plasma VLDL‐cholesterol and TAG, likely due to the high inclusion level and early dietary introduction, which promote hepatic lipid synthesis and transport. This response may have been further amplified by the indigestible polysaccharide matrix of untreated *C. vulgaris* and its high content of long‐chain polyunsaturated fatty acids, which can favour lipid mobilisation and VLDL production (Calder 2015; Calder 2017; Ebrahimi‐Mameghani et al. 2014; Karima and Sarto 2019; Lee et al. 2008; Shepherd 1989). Pancreatin superdosing (CVEN) selectively lowered HDL‐cholesterol, suggesting altered lipid partitioning via enhanced lipid digestion and hepatic flux, with higher circulating lipids but slightly lower hepatic lipid content, indicating increased mobilisation rather than storage (Barto et al. 2022; Hu et al. 2018).

Total plasma cholesterol was lower in CTR and CVEN birds, likely reflecting redistribution among lipoprotein fractions rather than a true hypocholesterolemic effect of dietary fibre and bioactive compounds (Cherng and Shih 2005; Hung et al. 2009; Perna et al. 2016; Ryu et al. 2014). LDL‐cholesterol was highest in CVEX potentially due to extrusion‐induced disruption of the microalgal matrix, enhancing lipid digestibility and hepatic export (Costa et al. 2024a; Mironeasa et al. 2023; Wang et al. 2018).

Total plasma protein was slightly reduced in CVEN birds, likely reflecting enhanced proteolysis and amino acid utilisation induced by enzyme superdosing, without impact on renal function or growth performance. CRP levels varied with diet but remained below physiological thresholds, indicating the absence of systemic inflammation. However, the lack of well‐established reference ranges limits its reliability as a standalone inflammatory biomarker in birds (Burkhardt et al. 2019; Seifi et al. 2014). Plasma electrolytes remained within physiological ranges, supporting homeostasis (Mushtaq and Pasha 2013; Shrimanker and Bhattarai 2023).

Dietary inclusion of *C. vulgaris* at 20% increased hepatic cholesterol concentrations, consistent with previous studies using microalgae (Alwaleed et al. 2021; Mendes et al. 2026; Park et al. 2018; Spínola et al. 2024). However, total hepatic lipid levels remained unchanged, in agreement with Coelho et al. (2021) and Mendes et al. (2026), suggesting that microalgal lipids were efficiently metabolised rather than stored as neutral lipids. Importantly, all *C. vulgaris* diets markedly improved the hepatic fatty acid profile by significantly reducing the *n‐6/n‐3* PUFA ratio, primarily due to increased deposition of long‐chain *n‐3* PUFAs (LC *n‐3* PUFAs), a response previously reported for both *C. vulgaris* and Spirulina (Mendes et al. 2026; Spínola et al. 2024) and generally considered beneficial for human health (Simopoulos 2016).

Hepatic docosahexaenoic acid (DHA; 22:6*n‐3*) levels increased by approximately 40% in the CVEX group and nearly doubled in the CVEN animals, with intermediate values observed in CV broilers. Also, eicosapentaenoic acid (EPA; 20:5*n‐3*) and docosapentaenoic acid (DPA; 22:5*n‐3*), increased substantially in all *C. vulgaris* diets. The greater enrichment of LC *n‐3* PUFAs in the CVEN group likely reflects enhanced lipid bioavailability via enzyme‐facilitated cell wall degradation of *C. vulgaris* and improved broiler intestinal absorption of dietary α‐linolenic acid (ALA; 18:3*n‐3*). In contrast, extrusion of *C. vulgaris* may have partially reduced ALA availability through thermal‐induced lipid modifications. Increased levels of LC *n‐3* PUFAs are associated with anti‐inflammatory effects, modulation of lipid metabolism, and support of antioxidant defences, primarily through EPA and DHA (Alagawany et al. 2019; Calder 2015; Simopoulos 2008).

Hepatic α‐tocopherol concentrations increased in all microalga‐fed groups, likely reflecting both dietary *C. vulgaris* content and elevated antioxidant demand due to higher pigment and PUFA levels. As the main form of vitamin E, α‐tocopherol acts as a chain‐breaking antioxidant, supporting oxidative balance and immune function in poultry (Surai 2002; Surai et al. 2017; Surai et al. 2019). γ‐Tocopherol and γ‐tocotrienol were higher in the CV group and attenuated by processing (CVEX, CVEN), possibly due to thermal degradation or altered absorption. Although γ‐tocopherol is not considered a vitamin E source in feed formulation, it can reduce reactive nitrogen species and modulate immune and inflammatory responses (Korošec et al. 2017).

Hepatic β‐carotene and chlorophyll‐like pigments were elevated in broilers from all *C. vulgaris* dietary treatments, which is consistent with its known composition (Becker 2007; Pulz and Gross 2004) and their deposition across tissues has been reported previously (Cabrol et al. 2022; Mendes et al. 2026; Mendes et al. 2025), influencing tissue colour and highlighting their physiological relevance. *C. vulgaris* extrusion reduced the β‐carotene content of the diet, indicating partial degradation of this compound during processing. Nevertheless, this reduction in dietary β‐carotene did not result in lower hepatic deposition, as similar levels were observed among all treatments containing microalgae, suggesting that the extent of degradation was not sufficient to affect hepatic accumulation. These pigments act as antioxidants, modulate immune responses, and may protect hepatic tissues from oxidative damage (Gupta et al. 2021; Pérez‐Gálvez et al. 2020; Serra et al. 2021; Varzaru et al. 2024).

The hepatic macro‐ and micromineral concentrations remained relatively stable across experimental diets containing *C. vulgaris*, indicating preserved mineral homeostasis. Notable exceptions included phosphorus (P) and copper (Cu), which were significantly higher in the CVEN broilers compared with the control broilers. These increases are likely related to pancreatin‐mediated disruption of the microalgal matrix, enhancing the release and bioavailability of intracellular and organically bound minerals.

All *C. vulgaris* dietary treatments resulted in higher hepatic manganese (Mn) concentrations relative to the CTR treatment. Given the role of Mn as a cofactor in enzymes involved in energy metabolism, antioxidant defence (e.g., Mn‐superoxide dismutase) and skeletal development, this increase may be particularly relevant in fast‐growing broilers and suggests a potential contribution of *C. vulgaris* to metabolic resilience (Noetzold et al. 2020; Olgun 2017). Previous research by Mendes et al. (2026), reported no change in Mn levels at 15% inclusion. This discrepancy may reflect the higher inclusion level employed here, which provides greater substrate availability for enzymatic action and mineral release.

The PCA of the hepatic metabolites revealed a distinct separation between the CTR group and all *C. vulgaris*–based groups, in agreement with previous reports (Coelho et al. 2021; Mendes et al. 2026; Spínola et al. 2024). This pattern indicates that microalga inclusion *per se* is the dominant driver of hepatic metabolic modulation, irrespective of processing or enzyme supplementation. The clustering of all microalgae‐fed groups suggests a consistent hepatic metabolic response, likely associated with shifts in lipid‐related parameters and antioxidant compounds, which have been previously linked to microalgae dietary inclusion.

In contrast, PCA of plasma metabolites revealed a more distinct response, with extrusion and enzyme superdosing promoting distinct clustering among the microalgae‐based diets. This separation is consistent with the observed differences in lipid‐related parameters among treatments, particularly the increases in TAG and VLDL‐cholesterol and the redistribution of lipoprotein fractions indicating that circulating metabolites—particularly total lipids, TAG and VLDL‐cholesterol—are highly sensitive to dietary processing strategies, corroborating previous findings by Coelho et al. (2021), Spínola et al. (2024) and Mendes et al. (2026).

## 5 Conclusion

To the best of our knowledge, this study offers the first detailed assessment of including 20% *C. vulgaris* as a macronutrient source in broiler diets concerning growth performance, blood markers, and hepatic metabolism. The dietary inclusion of *C. vulgaris* alone at this level negatively affected growth performance, reaffirming the limitations associated with its direct use in poultry feed.

The addition of pancreatin effectively alleviated these effects, bringing final body weight to levels similar to those seen in CTR animals and improving certain plasma lipid parameters and the liver fatty acid profile, without negatively impacting blood profiles. Therefore, these findings suggest that enzyme supplementation can enhance nutrient utilisation and support high‐level inclusion of *C. vulgaris* in broiler diets.

Although this study focused on 20% inclusion, literature suggests that extrusion or enzyme treatments can also benefit lower inclusion levels (~5%–10%), improving digestibility and bioactive compound availability without impairing growth.

Overall, these findings indicate the potential for partially replacing soybean meal with *C. vulgaris*, thereby supporting more sustainable poultry feed formulations. Future research should establish standard reference values for CRP in poultry, optimise *C. vulgaris* inclusion strategies, including processing and enzymatic treatments, and assess long‐term economic, environmental, and product‐quality outcomes to fully realise the benefits of *C. vulgaris* dietary inclusion.

## Author Contributions

**Ana R. Mendes:** Data curation, Formal analysis, Investigation, Writing – original draft preparation, Writing – review and editing; **Cátia F. Martins:** Data curation, Investigation, Writing – review and editing; **Maria P. Spínola:** Data curation, Investigation, Formal analysis, Writing – review and editing; **Daniela F. P. Carvalho:** Data curation, Investigation, Writing – review and editing; **Obete Madacussengua:** Data curation, Investigation, Writing – review and editing; **Joana I. Ferreira:** Data curation, Investigation, Writing – review and editing; **Ana M. Fontes:** Data curation, Investigation, Writing – review and editing; **Rui M. A. Pinto:** Formal analysis, Writing – review and editing; **Miguel P. Mourato:** Formal analysis, Writing ‐ review and editing; **Adam Davis:** Writing – review and editing; **André M. de Almeida:** Writing – review and editing; **Madalena Lordelo:** Writing – review and editing; **José A. M. Prates:** Conceptualisation, Funding acquisition, Project administration, Writing – review and editing.

## Ethics Statement

The methodology of this study was thoroughly reviewed and approved to ensure compliance with both national and European Union ethical standards for animal research. The research protocols were first approved by the Animal Welfare Committee (**ORBEA**) of the School of Agriculture at the University of Lisbon. Final approval was granted by the Animal Care Committee of the Directorate‐General for Food and Veterinary (**DGAV**) in Lisbon, Portugal, under protocol code 0421/000/000/2022.

## Conflicts of Interest

The authors declare no conflicts of interest.

## Data Availability

All data generated or analysed throughout this study are presented within this published article. Additional datasets obtained during the experimental phase can be made available by the corresponding author upon reasonable request.

## References

[jpn70064-bib-0001] Alwaleed, E.A. , M. El‐Sheekh , M.M. Abdel‐Daim , and H. Saber . 2021. “Effects of Spirulina Platensis and Amphora Coffeaeformis as Dietary Supplements on Blood Biochemical Parameters, Intestinal Microbial Population, and Productive Performance in Broiler Chickens.” Environmental Science and Pollution Research 28: 1801–1811. 10.1007/s11356-020-10597-3. 32857306

[jpn70064-bib-0002] Abdel‐Wareth, A. A. A. , A. N. Williams , M. Salahuddin , S. Gadekar , and J. Lohakare . 2024. “Algae as an Alternative Source of Protein in Poultry Diets for Sustainable Production and Disease Resistance: Present Status and Future Considerations.” Frontiers in Veterinary Science 11: 2024. 10.3389/fvets.2024.1382163 PMC11041637 38659457

[jpn70064-bib-0003] Alagawany, M. , S. S. Elnesr , M. R. Farag , et al. 2019. “Omega‐3 and Omega‐6 Fatty Acids in Poultry Nutrition: Effect on Production Performance and Health.” Animals: An Open Access Journal From MDPI 9: 573. 31426600 10.3390/ani9080573 PMC6721126

[jpn70064-bib-0004] Alfaia, C. M. , J. M. Pestana , M. Rodrigues , et al. 2021. “Influence of Dietary *Chlorella vulgaris* and Carbohydrate‐Active Enzymes on Growth Performance, Meat Quality and Lipid Composition of Broiler Chickens.” Poultry Science 100: 926–937. 10.1016/j.psj.2020.11.034 PMC7858185 33518146

[jpn70064-bib-0005] Alhattab, M. , A. Kermanshahi‐Pour , and M. S.‐L. Brooks . 2019. “Microalgae Disruption Techniques for Product Recovery: Influence of Cell Wall Composition.” Journal of Applied Phycology 31: 61–88.

[jpn70064-bib-0006] AOAC . 2000. “Official Methods of Analysis.” In the Association of Official Analytical Chemists (17th Edition). Gaithersburg, MD, USA.

[jpn70064-bib-0007] Asare, E. , Z. Yang , H. Yang , and Z. Wang . 2022. “Evaluation of Dietary Pancreatin as an Exogenous Enzyme on Growth Performance, Gene Expression, Immunological Responses, Serum Immunoglobins, and Intestinal Morphology in Cockerels.” Journal of Applied Animal Research 50: 61–68.

[jpn70064-bib-0008] Barto, T. L. , C. F. Morency , N. Schaap , A. B. Patel , and D. J. Monticello . 2022. “Intestinal Absorption of Lipids Using a Pancreatic Enzyme‐Free Nutritional Supplement in Patients With Cystic Fibrosis: A Randomized, Double‐Blind, Crossover Pilot Trial.” Nutrients 14: 680. 35277038 10.3390/nu14030680 PMC8838800

[jpn70064-bib-0009] Becker, E. W. 2007. Microalgae: Biotechnology and Microbiology.

[jpn70064-bib-0010] Bromfield, J. I. , L. C. Hoffman , D. Horyanto , and E. A. Soumeh . 2021. “Enhancing Growth Performance, Organ Development, Meat Quality, and Bone Mineralisation of Broiler Chickens Through Multi‐Enzyme Super‐Dosing in Reduced Energy Diets.” Animals: An Open Access Journal From MDPI 11: 2791. 34679813 10.3390/ani11102791 PMC8532949

[jpn70064-bib-0011] Burkhardt, N. B. , S. Röll , A. Staudt , et al. 2019. “The Long Pentraxin PTX3 Is of Major Importance Among Acute Phase Proteins in Chickens.” Frontiers in Immunology 10: 2019. 30774632 10.3389/fimmu.2019.00124 PMC6367253

[jpn70064-bib-0012] Cabrol, M. B. , J. C. Martins , L. P. Malhão , et al. 2022. “Partial Replacement of Soybean Meal With *Chlorella vulgaris* in Broiler Diets Influences Performance and Improves Breast Meat Quality and Fatty Acid Composition.” Poultry Science 101: 101955. 10.1016/j.psj.2022.101955 PMC9207287 35709682

[jpn70064-bib-0013] Calder, P. C. 2015. “Marine omega‐3 Fatty Acids and Inflammatory Processes: Effects, Mechanisms and Clinical Relevance.” Biochimica et Biophysica Acta (BBA) ‐ Molecular and Cell Biology of Lipids 1851: 469–484. 25149823 10.1016/j.bbalip.2014.08.010

[jpn70064-bib-0014] Calder, P. C. 2017. “Omega‐3 Fatty Acids and Inflammatory Processes: From Molecules to Man.” Biochemical Society Transactions 45: 1105–1115. 28900017 10.1042/BST20160474

[jpn70064-bib-0015] Çelekli, A. , B. Özbal , and H. Bozkurt . 2024. “Challenges in Functional Food Products With the Incorporation of Some Microalgae.” Foods 13: 725. 38472838 10.3390/foods13050725 PMC10930668

[jpn70064-bib-0016] Chaves, A. A. M. , C. F. Martins , D. F. P. Carvalho , et al. 2021. “A Viewpoint on the Use of Microalgae as an Alternative Feedstuff in the Context of Pig and Poultry Feeding‐A Special Emphasis on Tropical Regions.” Tropical Animal Health and Production 53: 396. 34247303 10.1007/s11250-021-02800-5

[jpn70064-bib-0017] Cherng, J.‐Y. , and M.‐F. Shih . 2005. “Preventing Dyslipidemia by *Chlorella pyrenoidosa* in Rats and Hamsters After Chronic High Fat Diet Treatment.” Life Sciences 76: 3001–3013. 15850594 10.1016/j.lfs.2004.10.055

[jpn70064-bib-0018] Chia, S. R. , K. W. Chew , H. F. M. Zaid , D.‐T. Chu , Y. Tao , and P. L. Show . 2019. “Microalgal Protein Extraction From *Chlorella vulgaris* FSP‐E Using Triphasic Partitioning Technique With Sonication.” Frontiers in Bioengineering and Biotechnology 7: 396. 10.3389/fbioe.2019.00396. 31867321 PMC6908848

[jpn70064-bib-0019] Coelho, D. F. M. , C. M. R. P. M. Alfaia , J. M. P. Assunção , et al. 2021. “Impact of Dietary *Chlorella vulgaris* and Carbohydrate‐Active Enzymes Incorporation on Plasma Metabolites and Liver Lipid Composition of Broilers.” BMC Veterinary Research 17: 229. 34187475 10.1186/s12917-021-02932-8 PMC8243889

[jpn70064-bib-0020] Costa, M. M. , M. P. Spínola , V. D. Alves , and J. A. Mestre Prates . 2024a. “Improving Protein Extraction and Peptide Production From *Chlorella vulgaris* Using Combined Mechanical/Physical and Enzymatic Pre‐Treatments.” Heliyon 10: e32704. 38988577 10.1016/j.heliyon.2024.e32704 PMC11233943

[jpn70064-bib-0021] Costa, M. M. , M. P. Spínola , B. Tavares , et al. 2024b. “Effects of High Dietary Inclusion of Arthrospira Platensis, Either Extruded or Supplemented With a Super‐Dosing Multi‐Enzyme Mixture, on Broiler Growth Performance and Major Meat Quality Parameters.” BMC Veterinary Research 20: 176. 38711127 10.1186/s12917-024-04027-6 PMC11071269

[jpn70064-bib-0022] Covaci, A. , S. Voorspoels , C. Thomsen , B. van Bavel , and H. Neels . 2006. “Evaluation of Total Lipids Using Enzymatic Methods for the Normalization of Persistent Organic Pollutant Levels in Serum.” Science of the Total Environment 366: 361–366. 16624383 10.1016/j.scitotenv.2006.03.006

[jpn70064-bib-0023] Dinalli, V. , A. Soares , R. Carvalho , et al. 2024. “Carcass Characteristics and Meat Quality of Broiler Chickens Fed Diets With *Chlorella vulgaris* and Probiotic.” Brazilian Journal of Poultry Science 26. 10.1590/1806-9061-2024-1937.

[jpn70064-bib-0024] dos Santos Madeira, M. S. M. , P. A. A. B. Lopes , C. F. Martins , et al. 2021. “Dietary Arthrospira Platensis Improves Systemic Antioxidant Potential and Changes Plasma Lipids Without Affecting Related Hepatic Metabolic Pathways in Post‐Weaned Piglets.” BMC Veterinary Research 17: 158. 33849543 10.1186/s12917-021-02869-y PMC8045302

[jpn70064-bib-0025] Ebrahimi‐Mameghani, M. , S. Aliashrafi , Y. Javadzadeh , and M. AsghariJafarabadi . 2014. “The Effect of *Chlorella vulgaris* Supplementation on Liver En‐zymes, Serum Glucose and Lipid Profile in Patients With Non‐Alcoholic Fatty Liver Disease.” Health Promotion Perspectives 4: 107–115. 25097844 10.5681/hpp.2014.014 PMC4122038

[jpn70064-bib-0026] El‐Ghany, W. A. A. 2020. “Microalgae in Poultry Field: A Comprehensive Perspectives.” Advances in Animal and Veterinary Sciences 8: 888–897.

[jpn70064-bib-0027] El‐Shall, N. A. , S. Jiang , M. R. Farag , et al. 2023. “Potential of *Spirulina platensis* as a Feed Supplement for Poultry to Enhance Growth Performance and Immune Modulation.” Frontiers in Immunology 14: 1072787. 36798131 10.3389/fimmu.2023.1072787 PMC9927202

[jpn70064-bib-0028] Fernandes, E. A. , C. F. Martins , and J. R. Sales , et al. 2024. “Impact of a 15% Spirulina (*Limnospira platensis*) Dietary Inclusion on Productive Performance and Meat Traits in Naked Neck and Fully Feathered Slow‐Growing Broiler Strains.” Poultry Science 103: 104106. 10.1016/j.psj.2024.104106 PMC11381824 39159573

[jpn70064-bib-0029] Folch, J. , M. Lees , and G. H. S. Stanley . 1957. “A Simple Method for the Isolation and Purification of Total Lipides From Animal Tissues.” Journal of Biological Chemistry 226: 497–509. 13428781

[jpn70064-bib-0030] Friedewald, W. T. , R. I. Levy , and D. S. Fredrickson . 1972. “Estimation of the Concentration of Low‐Density Lipoprotein Cholesterol in Plasma, Without Use of the Preparative Ultracentrifuge.” Clinical Chemistry 18: 499–502. 4337382

[jpn70064-bib-0031] Gerken, H. G. , B. Donohoe , and E. P. Knoshaug . 2013. “Enzymatic Cell Wall Degradation of *Chlorella vulgaris* and Other Microalgae for Biofuels Production.” Planta 237: 239–253. 23011569 10.1007/s00425-012-1765-0

[jpn70064-bib-0032] Gupta, A. K. , K. Seth , K. Maheshwari , et al. 2021. “Biosynthesis and Extraction of High‐Value Carotenoid From Algae.” Frontiers in Bioscience‐Landmark 26: 171–190. 10.52586/4932 34162044

[jpn70064-bib-0033] Hu, Y. D. , D. Lan , Y. Zhu , H. Z. Pang , X. P. Mu , and X. F. Hu . 2018. “Effect of Diets With Different Energy and Lipase Levels on Performance, Digestibility and Carcass Trait in Broilers.” Asian‐Australasian Journal of Animal Sciences 31: 1275–1284. 29268569 10.5713/ajas.17.0755 PMC6043444

[jpn70064-bib-0034] Hung, S.‐C. , G. Bartley , S. A. Young , et al. 2009. “Dietary Fiber Improves Lipid Homeostasis and Modulates Adipocytokines in Hamsters.” Journal of Diabetes 1: 194–206. 20923539 10.1111/j.1753-0407.2009.00034.x

[jpn70064-bib-0035] Hynstova, V. , D. Sterbova , B. Klejdus , J. Hedbavny , D. Huska , and V. Adam . 2018. “Separation, Identification and Quantification of Carotenoids and Chlorophylls in Dietary Supplements Containing *Chlorella vulgaris* and *Spirulina platensis* Using High Performance Thin Layer Chromatography.” Journal of Pharmaceutical and Biomedical Analysis 148: 108–118. 28987995 10.1016/j.jpba.2017.09.018

[jpn70064-bib-0036] Kang, H. K. , H. M. Salim , N. Akter , et al. 2013. “Effect of Various Forms of Dietary Chlorella Supplementation on Growth Performance, Immune Characteristics, and Intestinal Microflora Population of Broiler Chickens.” Journal of Applied Poultry Research 22: 100–108.

[jpn70064-bib-0037] Karima, F. N. , and M. Sarto . 2019. “The Effect of *Chlorella vulgaris* on Lipid Profile Wistar Strain Rats (*Rattus norvegicus berkenhout, 1769*) Under Induced Stress.” Biogenesis: Jurnal Ilmiah Biologi 7: 44.

[jpn70064-bib-0038] Korošec, T. , U. Tomažin , S. Horvat , R. Keber , and J. Salobir . 2017. “The Diverse Effects of α‐ and γ‐tocopherol on Chicken Liver Transcriptome.” Poultry Science 96: 667–680. 10.3382/ps/pew296 27587731

[jpn70064-bib-0039] Lee, H. S. , H. J. Park , and M. K. Kim . 2008. “Effect of *Chlorella vulgaris* on Lipid Metabolism in Wistar Rats Fed High Fat Diet.” Nutrition Research and Practice 2: 204–210. 20016720 10.4162/nrp.2008.2.4.204 PMC2788184

[jpn70064-bib-0040] Lee, J. Y. , J. H. Yoon , S. H. An , et al. 2023. “Intestinal Immune Cell Populations, Barrier Function, and Microbiomes in Broilers Fed a Diet Supplemented With *Chlorella vulgaris* .” Animals: An Open Access Journal From MDPI 13: 2380. 37508157 10.3390/ani13142380 PMC10376636

[jpn70064-bib-0041] Machado, L. , G. Carvalho , and R. N. Pereira . 2022. “Effects of Innovative Processing Methods on Microalgae Cell Wall: Prospects Towards Digestibility of Protein‐Rich Biomass.” Biomass 2: 80–102.

[jpn70064-bib-0042] Madacussengua, O. , A. R. Mendes , C. F. Martins , D. Carvalho , A. Md Almeida , and M. Lordelo . 2024. “The Effects of Replacing Soybean Meal With *Chlorella vulgaris* in Laying Hen Diets on Performance and Physical Characteristics of Eggs.” Animals: An Open Access Journal From MDPI 14: 2552. 39272337 10.3390/ani14172552 PMC11394650

[jpn70064-bib-0043] Madigan‐Stretton, J. , D. Mikkelsen , and E. A. Soumeh . 2021. “Multienzyme Super‐Dosing in Broiler Chicken Diets: The Implications for Gut Morphology, Microbial Profile, Nutrient Digestibility, and Bone Mineralization.” Animals: An Open Access Journal From MDPI 11, no. 1: 1. 10.3390/ani11010001 PMC7821924 33374896

[jpn70064-bib-0044] Martins, C. F. , P. A. Lopes , M. Palma , et al. 2022. “Impact of Dietary *Chlorella vulgaris* and Feed Enzymes on Health Status, Immune Response and Liver Metabolites in Weaned Piglets.” Scientific Reports 12: 16816. 36207385 10.1038/s41598-022-21238-9 PMC9546893

[jpn70064-bib-0045] Mendes, A. R. , O. Madacussengua , C. F. Martins , et al. 2026. “High Inclusion of *Chlorella vulgaris* in Broiler Diets Improves Feed Efficiency and Liver n‑3 Fatty Acid Profile: Effects of Extrusion and Enzyme Super‐Dosing.” British Poultry Science 12: 1–15. 10.1080/00071668.2025.2606838 41524449

[jpn70064-bib-0046] Mendes, A. R. , O. Madacussengua , J. M. Pestana , et al. 2025. “High Dietary Inclusion of Chlorella Vulgaris With Extrusion or Enzyme Superdosing: Effects on Broiler Performance, Welfare, and Meat Quality.” Poultry Science 104: 105372. 10.1016/j.psj.2025.105372 PMC12173054 40480138

[jpn70064-bib-0047] Mendes, A. R. , M. P. Spínola , M. Lordelo , and J. A. M. Prates . 2024a. “Assessing the Influence of Cumulative *Chlorella vulgaris* Intake on Broiler Carcass Traits, Meat Quality and Oxidative Stability.” Foods 13: 2753. 39272518 10.3390/foods13172753 PMC11395549

[jpn70064-bib-0048] Mendes, A. R. , M. P. Spínola , M. Lordelo , and J. A. M. Prates . 2024b. “Impact of *Chlorella vulgaris* Intake Levels on Performance Parameters and Blood Health Markers in Broiler Chickens.” Veterinary Sciences 11: 290. 39057974 10.3390/vetsci11070290 PMC11281427

[jpn70064-bib-0049] Merin, C. T. , S. J. Bunglavan , B. Chako , S. Senthil Murugan , and P. B. Aswathi . 2022. “Effect of Varied Levels of Dietary *Chlorella vulgaris* Extract on Blood Biochemical Profile and Immune Organ Status in Broilers.” Journal of Veterinary and Animal Sciences 53: 435–440.

[jpn70064-bib-0050] Mestre Prates, J. A. , M. A. Gonçalves Quaresma , R. J. Branquinho Bessa , C. M. G. Andrade Fontes , and C. M. P. Mateus Alfaia . 2006. “Simultaneous HPLC Quantification of Total Cholesterol, Tocopherols and β‐Carotene in Barrosã‐PDO veal.” Food Chemistry 94: 469–477.

[jpn70064-bib-0051] Mironeasa, S. , I. Coţovanu , C. Mironeasa , and M. Ungureanu‐Iuga . 2023. “A Review of the Changes Produced by Extrusion Cooking on the Bioactive Compounds From Vegetal Sources.” Antioxidants 12: 1453. 37507991 10.3390/antiox12071453 PMC10376774

[jpn70064-bib-0052] Mirzaie, S. , F. Zirak‐Khattab , S. A. Hosseini , and H. Donyaei‐Darian . 2018. “Effects of Dietary Spirulina on Antioxidant Status, Lipid Profile, Immune Response and Performance Characteristics of Broiler Chickens Reared Under High Ambient Temperature.” Asian‐Australasian Journal of Animal Sciences 31: 556–563. 28920419 10.5713/ajas.17.0483 PMC5838328

[jpn70064-bib-0053] Mushtaq, M. M. H. , and T. N. Pasha . 2013. “Electrolytes, Dietary Electrolyte Balance and Salts in Broilers: Anupdated Review on Acid‐Base Balance, Blood and Carcass Characteristics.” World's Poultry Science Journal 69: 833–852.

[jpn70064-bib-0054] Noetzold, T. L. , S. L. Vieira , A. Favero , R. M. Horn , C. M. Silva , and G. B. Martins . 2020. “Manganese Requirements of Broiler Breeder Hens.” Poultry Science 99: 5814–5826. 10.1016/j.psj.2020.06.085 PMC7647800 33142499

[jpn70064-bib-0055] Olgun, O. 2017. “Manganese in Poultry Nutrition and Its Effect on Performance and Eggshell Quality.” World's Poultry Science Journal 73: 45–56.

[jpn70064-bib-0056] Park, J. H. , S. I. Lee , and I. H. Kim . 2018. “Effect of Dietary *Spirulina (Arthrospira) platensis* on the Growth Performance, Antioxidant Enzyme Activity, Nutrient Digestibility, Cecal Microflora, Excreta Noxious Gas Emission, and Breast Meat Quality of Broiler Chickens.” Poultry Science 97: 2451–2459. 10.3382/ps/pey093 29672750

[jpn70064-bib-0057] Pérez‐Gálvez, A. , I. Viera , and M. Roca . 2020. “Carotenoids and Chlorophylls as Antioxidants.” Antioxidants 9: 505. 32526968 10.3390/antiox9060505 PMC7346216

[jpn70064-bib-0058] Perna, S. , A. Giacosa , G. Bonitta , et al. 2016. “Effects of Hazelnut Consumption on Blood Lipids and Body Weight: A Systematic Review and Bayesian Meta‐Analysis.” Nutrients 8: 747. 27897978 10.3390/nu8120747 PMC5188407

[jpn70064-bib-0059] Pestana, J. M. , B. Puerta , H. Santos , et al. 2020. “Impact of Dietary Incorporation of Spirulina (*Arthrospira platensis*) and Exogenous Enzymes on Broiler Performance, Carcass Traits, and Meat Quality.” Poultry Science 99: 2519–2532. 10.1016/j.psj.2019.11.069 PMC7597389 32359588

[jpn70064-bib-0060] Pulz, O. , and W. Gross . 2004. “Valuable Products From Biotechnology of Microalgae.” Applied Microbiology and Biotechnology 65: 635–648. 15300417 10.1007/s00253-004-1647-x

[jpn70064-bib-0061] Ribeiro, D. M. , T. Scanlon , T. Kilminster , et al. 2020. “Mineral Profiling of Muscle and Hepatic Tissues of Australian Merino, Damara and Dorper Lambs: Effect of Weight Loss.” Journal of Animal Physiology and Animal Nutrition 104: 823–830. 32166799 10.1111/jpn.13339

[jpn70064-bib-0062] Ryu, N. H. , Y. Lim , J. E. Park , et al. 2014. “Impact of Daily Chlorella Consumption on Serum Lipid and Carotenoid Profiles in Mildly Hypercholesterolemic Adults: A Double‐Blinded, Randomized, Placebo‐Controlled Study.” Nutrition Journal 13: 57. 24920270 10.1186/1475-2891-13-57 PMC4066283

[jpn70064-bib-0063] Seifi, S. , S. H. Alian Samakkhah , and K. Absalan Fard . 2017. “Acute Phase Response in Experimentally Infected Broilers With Avian Infectious Bronchitis Virus Serotype 4/91.” Journal of the Hellenic Veterinary Medical Society 65: 17–22.

[jpn70064-bib-0064] Serra, A. T. , S. D. Silva , L. Pleno de Gouveia , et al. 2021. “A Single Dose of Marine *Chlorella vulgaris* Increases Plasma Concentrations of Lutein, β‐Carotene and Zeaxanthin in Healthy Male Volunteers.” Antioxidants 10: 1164. 34439412 10.3390/antiox10081164 PMC8388909

[jpn70064-bib-0065] Shepherd, J. 1989. “Mechanism of Action of Bile Acid Sequestrants and Other Lipid‐Lowering Drugs.” Cardiology 76, no. Suppl 1: 65–74. 2713876 10.1159/000174548

[jpn70064-bib-0066] Shrimanker, I. , and S. Bhattarai . 2023. Electrolytes. Treasure Island (FL): StatPearls Publishing. 31082167

[jpn70064-bib-0067] Simopoulos, A. 2016. “An Increase in the Omega‐6/Omega‐3 Fatty Acid Ratio Increases the Risk for Obesity.” Nutrients 8: 128. 26950145 10.3390/nu8030128 PMC4808858

[jpn70064-bib-0068] Simopoulos, A. P. 2008. “The Importance of the Omega‐6/Omega‐3 Fatty Acid Ratio in Cardiovascular Disease and Other Chronic Diseases.” Experimental Biology and Medicine 233: 674–688. 18408140 10.3181/0711-MR-311

[jpn70064-bib-0069] Spínola, M. P. , C. M. Alfaia , M. M. Costa , et al. 2024. “Impact of High Spirulina Diet, Extruded or Supplemented With Enzymes, on Blood Cells, Systemic Metabolites, and Hepatic Lipid and Mineral Profiles of Broiler Chickens.” Frontiers in Veterinary Science 11: 1342310. 38596464 10.3389/fvets.2024.1342310 PMC11002084

[jpn70064-bib-0070] Spínola, M. P. , M. M. Costa , and J. A. M. Prates . 2023a. “Effect of Selected Mechanical/Physical Pre‐Treatments on *Chlorella vulgaris* Protein Solubility.” Agriculture (London) 13: 1309.

[jpn70064-bib-0071] Spínola, M. P. , M. M. Costa , and J. A. M. Prates . 2023b. “Enhancing Digestibility of *Chlorella vulgaris* Biomass in Monogastric Diets: Strategies and Insights.” Animals: An Open Access Journal From MDPI 13: 1017. 36978557 10.3390/ani13061017 PMC10044532

[jpn70064-bib-0072] Surai, P. 2002. Natural Antioxidants in Avian Nutrition and Reproduction, 5–9. Nottingham University Press.

[jpn70064-bib-0073] Surai, P. F. , I. I. Kochish , and V. I. Fisinin . 2017. “Antioxidant Systems in Poultry Biology: Nutritional Modulation of Vitagenes.” European Poultry Science 81: 1–21.

[jpn70064-bib-0074] Surai, P. F. , I. I. Kochish , M. N. Romanov , and D. K. Griffin . 2019. “Nutritional Modulation of the Antioxidant Capacities in Poultry: The Case of Vitamin E.” Poultry Science 98: 4030–4041. 10.3382/ps/pez072 30805637

[jpn70064-bib-0075] Vale, M. M. , D. R. Klein , T. Branco , and M. P. Santos . 2019. “Glycemic Response of Poultries in Different Feeding Systems.” Acta Scientiarum. Animal Sciences 41: 43148.

[jpn70064-bib-0076] Vandamme, D. , I. Foubert , and K. Muylaert . 2013. “Flocculation as a Low‐Cost Method for Harvesting Microalgae for Bulk Biomass Production.” Trends in Biotechnology 31: 233–239. 23336995 10.1016/j.tibtech.2012.12.005

[jpn70064-bib-0077] Van Nerom, S. , K. Buyse , F. Van Immerseel , J. Robbens , and E. Delezie . 2024. “Pulsed Electric Field (PEF) Processing of Microalga *Chlorella vulgaris* and Its Digestibility in Broiler Feed.” Poultry Science 103: 103721. 10.1016/j.psj.2024.103721 PMC11636916 38613915

[jpn70064-bib-0078] Varzaru, I. , A. E. Untea , T. D. Panaite , et al. 2024. “ *Chlorella vulgaris* as a Nutraceutical Source for Broilers: Improving Meat Quality and Storage Oxidative Status.” Foods 13: 2373. 39123564 10.3390/foods13152373 PMC11312065

[jpn70064-bib-0079] Wang, M. , H. Cheng , S. Chen , et al. 2018. “Microalgal Cell Disruption via Extrusion for the Production of Intracellular Valuables.” Energy 142: 339–345.

[jpn70064-bib-0080] Weber, S. , P. M. Grande , L. M. Blank , and H. Klose . 2022. “Insights Into Cell Wall Disintegration of *Chlorella vulgaris* .” PLoS One 17: e0262500. 35030225 10.1371/journal.pone.0262500 PMC8759652

[jpn70064-bib-0081] Xing, M. , M. Gao , J. Li , P. Han , L. Mei , and L. Zhao . 2022. “Characteristics of Peripheral Blood Gamma‐Glutamyl Transferase in Different Liver Diseases.” Medicine 101: e28443. 35029891 10.1097/MD.0000000000028443 PMC8735790

[jpn70064-bib-0082] Yongabi Anchang, K. , D. Lewis , and C. Nji . 2016. “Toxicological, Phytochemical, and Antibacterial Assessment of *Chlorella vulgaris* and *Spirulina platensis* Poder in Albino Rats. a Preliminary Study.” Revista Peruana de Medicina Integrativa 1: 5–11.

[jpn70064-bib-0083] Zampiga, M. , G. Brugaletta , F. Ceccaroni , A. Bonaldo , S. Pignata , and F. Sirri . 2023. “Performance Response of Broiler Chickens Fed Diets Containing Dehydrated Microalgae Meal as Partial Replacement for Soybean Until 22 Days of Age.” Animal Feed Science and Technology 297: 115573.

[jpn70064-bib-0084] Zampiga, M. , L. Laghi , F. Soglia , et al. 2024. “Partial Substitution of Soybean Meal With Microalgae Meal (*Arthrospira* spp. – Spirulina) in Grower and Finisher Diets for Broiler Chickens: Implications on Performance Parameters, Footpad Dermatitis Occurrence, Breast Meat Quality Traits, Amino Acid Digestibility and Plasma Metabolomics Profile.” Poultry Science 103, no. 8: 103856. 10.1016/j.psj.2024.103856 PMC11253657 38908124

[jpn70064-bib-0085] Zhang, L. , Y. Jiang , J. A. Buzdar , et al. 2025. “Microalgae: An Exciting Alternative Protein Source and Nutraceutical for the Poultry Sector.” Food Science of Animal Resources 45: 243–265. 39840237 10.5851/kosfa.2024.e130 PMC11743838

